# Estimating chorusing activity by quantifying total acoustic energy

**DOI:** 10.1121/10.0013351

**Published:** 2022-08-02

**Authors:** Andrea Megela Simmons, Chen Ming, Laura N. Kloepper

**Affiliations:** 1 Department of Cognitive, Linguistic, & Psychological Sciences, Brown University, Providence, Rhode Island, 02912, USA; 2 Carney Institute for Brain Science, Brown University, Providence, Rhode Island 02912, USA; 3 Department of Biological Sciences, College of Life Sciences and Agriculture, University of New Hampshire, Durham, New Hampshire 03824, USA Andrea_Simmons@brown.edu, chen_ming@brown.edu, laura.kloepper@unh.edu

## Abstract

Passive acoustics provides a powerful method for localizing vocalizing animals and estimating species abundance. A passive acoustics method previously used to census dense populations of flying bats is applied here to estimate chorusing activity of male bullfrogs vocalizing against anthropogenic noise. There are significant links between manual counts of the numbers of advertisement call notes and automatically detected notes and two measures of acoustic energy. These data provide a foundation for the use of acoustic energy measures to census vocal activity in different habitats.

## 1. Introduction

Bioacoustic monitoring provides an important metric to evaluate environmental impacts, including climate change, on species behavior, survival, and abundance (Kloepper and Simmons, 2014; Kvsn *et al.*, 2020). Identifying individual species vocalizations in the natural environment is challenging, however, because animals often vocalize in groups in the midst of biological and anthropogenic noise sources that can interfere with their own perception of sounds as well as an observer's ability to document and count these sounds. In many acoustic surveys, vocalizations of sequential focal (target) animals are counted by a trained listener or automated detection by a computer algorithm, preceded or followed by noise reduction techniques to isolate the signal of interest from background noise (Aide *et al.*, 2013; Kvsn *et al.*, 2020; Sainburg and Gentner, 2021). Other methods (Pieretti *et al.*, 2011; Sueur *et al.*, 2008) census groups of vocalizing animals, such as choruses of singing birds or of calling frogs, where instead of identifying a specific focal animal, the entire soundscape is recorded simultaneously.

Kloepper *et al.* (2016) recently introduced a method based on overall acoustic energy to census flying echolocating bats. This initial approach recorded echolocation calls emitted by dense groups of Brazilian free-tailed bats (*Tadarida brasiliensis*) emerging at dusk from a fixed point (i.e., their roost), flying at a uniform distance past a single sensor located at a fixed position. This energy measure eliminated the need to detect and isolate signals from individual bats. Because bats emit ultrasonic signals, far above the frequency range of common anthropogenic noise sources, recordings were not contaminated by this excess noise. Many animals, however, emit vocalizations that fall within the frequency range of interfering noise sources such as vehicles. Isolating these vocalizations from noise can be challenging (Pieretti *et al.*, 2011; Sainburg and Gentner, 2021). Here, we test whether the energy detection algorithm can be used to estimate vocal activity of small groups of chorusing bullfrogs living in a habitat exposed to anthropogenic noise as well as acoustic interference from other vocalizing frog species.

In their breeding season, male bullfrogs (*Rana catesbeiana*) form choruses in which they emit harmonically structured, multiple-note advertisement calls to announce occupation of a calling site to rival males and to attract females for mating (Capranica, 1965; Emlen, 1976; Howard, 1978). Bullfrog ponds are located in areas exposed to various levels of human-generated noise (traffic, construction, jets) and to sounds of other vocal species; for effective communication, both callers and listeners must contend with these noise sources. Males often call simultaneously or partially overlapping with their calling neighbors, making manual human isolation of individual callers challenging (Bates *et al.*, 2010). To investigate the usefulness of the energy detection measure for censusing non-echolocating species, we reanalyzed four spontaneous choruses of male bullfrogs where the numbers of individual callers and their locations were known. We investigated the link between number of individual notes, quantified manually, and two measures of acoustic energy quantified by our algorithm. Our data suggest that the energy detection method can be used to estimate the numbers of vocalizations and possibly the abundance of vocalizing frogs communicating in the midst of anthropogenic noise.

## 2. Methods

We reanalyzed data previously recorded (Boatright-Horowitz *et al.*, 2000; Suggs and Simmons, 2005; Bates *et al.*, 2010) from a small pond in a residential neighborhood in eastern Rhode Island. The pond is under a flight path from a local airport, so jet noise was common; traffic and other anthropogenic noise sources were also present. True noise levels of these background sources were not quantified at the time of recordings. Small numbers of green frogs (*Rana clamitans*) also inhabited the pond; these frogs called sporadically during times of bullfrog chorusing, using short vocalizations readily distinguishable by human listeners from the longer duration notes emitted by bullfrogs (Bee and Perrill, 1996; Simmons, 2004). Before recording sessions began, the spatial positions of all calling male bullfrogs were visualized using flashlights and marked on a map of the pond. Males typically remained in and around their own calling spots, with little to no movement to other locations once chorusing commenced. Numbers of non-vocalizing males, which were less likely to remain stationary, and of females could not be ascertained.

In 1994, spontaneous chorusing was recorded for 95 (070294; 8 calling males), 45 (070394; 7 males), and 80 (070894; 6 males) min with a Sennheiser ME66 microphone (frequency response 0.05–20 kHz, +/−3 dB;) onto a Marantz model PMD430 cassette recorder (frequency response 0.05–14 kHz, +/−3 dB). Recordings on 061205 (6 calling males) were made for 90 min using four Knowles electret condenser microphones arranged in a sensor array (described in Bates *et al.*, 2010). We digitized all of the field recordings using Adobe Audition 2020 (sampling rate 44 100 Hz, 16 bit). Each of the digitized recordings were then segmented manually (time selection tool in Adobe Audition) into 5-min segments, providing 19, 9, 16, and 18 samples per night, respectively, for analysis. A trained observer (the first author) aurally and visually identified (on the basis of harmonic structure shown in the spectral frequency display computed with Adobe Audition, sampling rate 16 000 Hz), and counted all individual bullfrog notes in each 5-min segment regardless of note amplitude (i.e., the bullfrog's distance from the recording microphone).

**Fig. 1.**
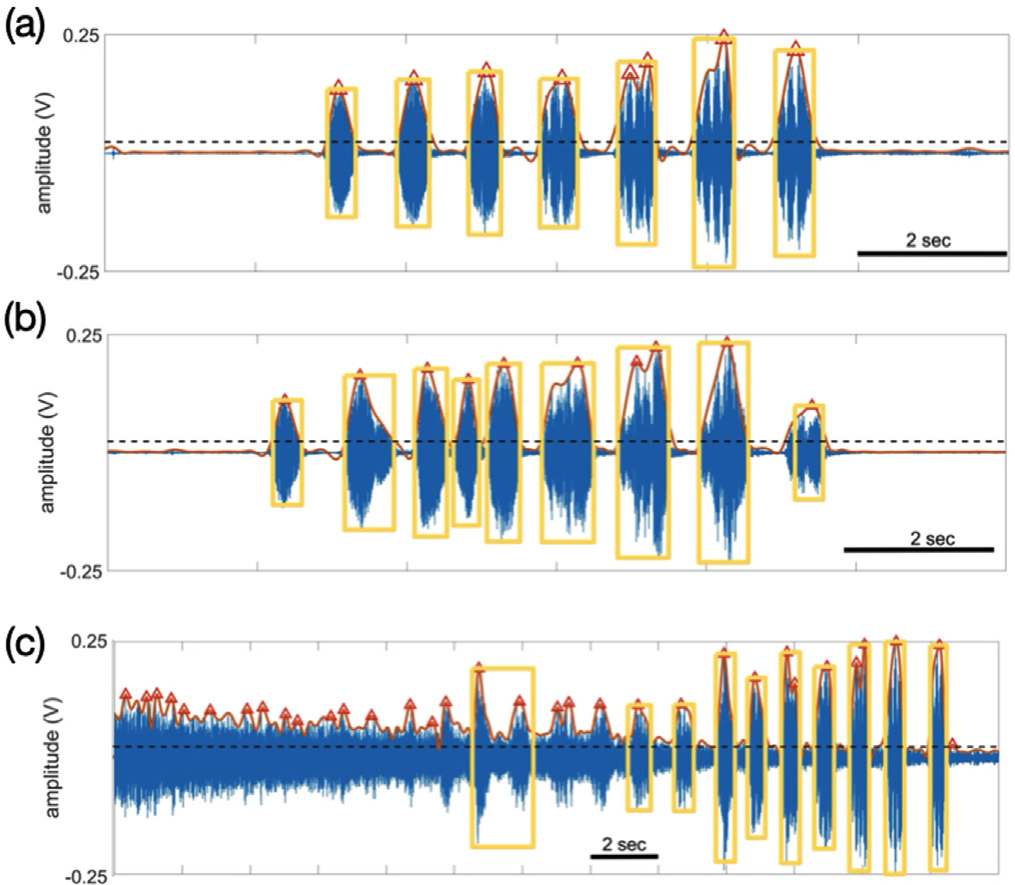
Examples of the detected note algorithm. (a) Detected notes (yellow bounding boxes) of a single bullfrog calling. This advertisement call consists of seven individual notes. Red triangles mark the peak of each detected envelope; these peaks were used to calculate the number of automatically detected notes. The detected note acoustic energy was calculated by adding the squares of the amplitudes in the blue waveform within each yellow bounding box. (b) Detected notes of two bullfrogs calling, producing overlapping notes. The second bounding box covers two notes from the two frogs. (c) Example of detected frog calls in the presence of overlapping jet noise. Two bullfrog notes, identified aurally and by visual inspection of spectrograms, immediately after the left-most bounding box were not detected by the algorithm. Red triangles not within bounding boxes (i.e., those prior to the left-most bounding box) are masked by noise with amplitudes higher than the threshold, and the bounding box lengths are longer than 2 s. These red triangles could denote either bullfrog notes or background noise. Three bullfrogs were calling in this example, as shown by different note amplitudes. Data from 061205.

For automated analysis, we identified parts of each digitized recording containing jet noise to construct a noise print in Adobe Audition using the Noise Reduction algorithm. This noise print was then applied to the entire audio file to reduce these background noises. The recording was then passed through a Butterworth filter with a cut-off frequency of 2000 Hz, the upper frequency limit of harmonics in bullfrog advertisement calls (Capranica, 1965; Bee, 2004). A custom script written in matlab 2021a (MathWorks, Natick MA) was developed to detect individual bullfrog notes (Fig. 1) and to calculate acoustic energy (Kloepper *et al.*, 2016) using two approaches: (a) total acoustic energy and (b) detected note acoustic energy. The total acoustic energy (in V^2^) in each noise-reduced, filtered 5-min sample was calculated by the sum of squares of the waveform, while the detected note acoustic energy (in V^2^) was determined by adding the squares of amplitudes in the bounding boxes that the in-house matlab script detected around each note, or overlapping notes. Examples of the second, note detection approach to calculate the detected note acoustic energy are as follows and depicted in Fig. 1.

Individual notes are picked in two stages. First, the recordings were processed through a bandpass filter with a passband between 1.5 and 2 kHz and stop band attenuation 65 dB at frequencies 1 and 2.5 kHz; the filter was an equiripple FIR filter using Parks-McClellan algorithm (Parks and McClellan, 1972), where the passband ripple was 0.5 dB. Equiripple filters have a frequency response that minimizes the maximum ripple magnitude over all bands. The distribution of the amplitudes in the filtered waveform was fit with a normal function, where twice the standard deviation was used as a threshold for determining peaks. The minimum time interval between neighboring peaks was 20 ms. The second stage identified bullfrog notes among peaks determined from stage 1. The program searched both left and right directions until the absolute amplitudes in a window with length 500 of the filtered waveform were all below the threshold. The number 500 was used to pinpoint the boundary where the threshold intercepts each note, while longer or shorter windows might miss the boundary. Bullfrog advertisement notes have durations of 400–700 ms (Simmons, 2004); green frog advertisement calls are considerably shorter, about 200 ms long (Bee and Perrill, 1996). Bullfrog notes were then identified by bounding box widths longer than 0.2 s, meant to exclude very brief noise bursts and the shorter calls of green frogs. The bounding box had an upper limit of 2 s, selected to capture overlapping notes that result in wider bounding boxes (Fig. 1) while excluding overlapping wideband noise, such as that produced by jets flying over the pond.

To investigate the link between frog notes and acoustic energy, we used linear regression analysis with number of manually counted notes as the predictor variable and both total acoustic energy and detected note acoustic energy, as defined above, as the response variables. We also calculated the regression between manually counted and automatically detected notes (all notes detected and marked with a red triangle within bounding boxes; Fig. 1). All statistics were calculated in IBM SPSS v28.

## 3. Results

Bullfrog vocal activity varied over the four recording nights, producing a variable number of advertisement call notes. We found significant relationships between numbers of manually counted and automatically detected notes for all recording nights (Table 1), although the strength of the r^2^ value varied.

**TABLE 1.** Relation between manually counted and automatically detected call notes on each recording night.

Date	Length (min)	Number of males	Total manual notes	Total automatic notes	r^2^	p
070294	95	8	2967	1639	0.209	0.049
070394	45	7	826	1548	0.733	0.003
070894	80	6	895	760	0.643	0.0001
061205	90	6	1376	1350	0.402	0.005

**Fig. 2.**
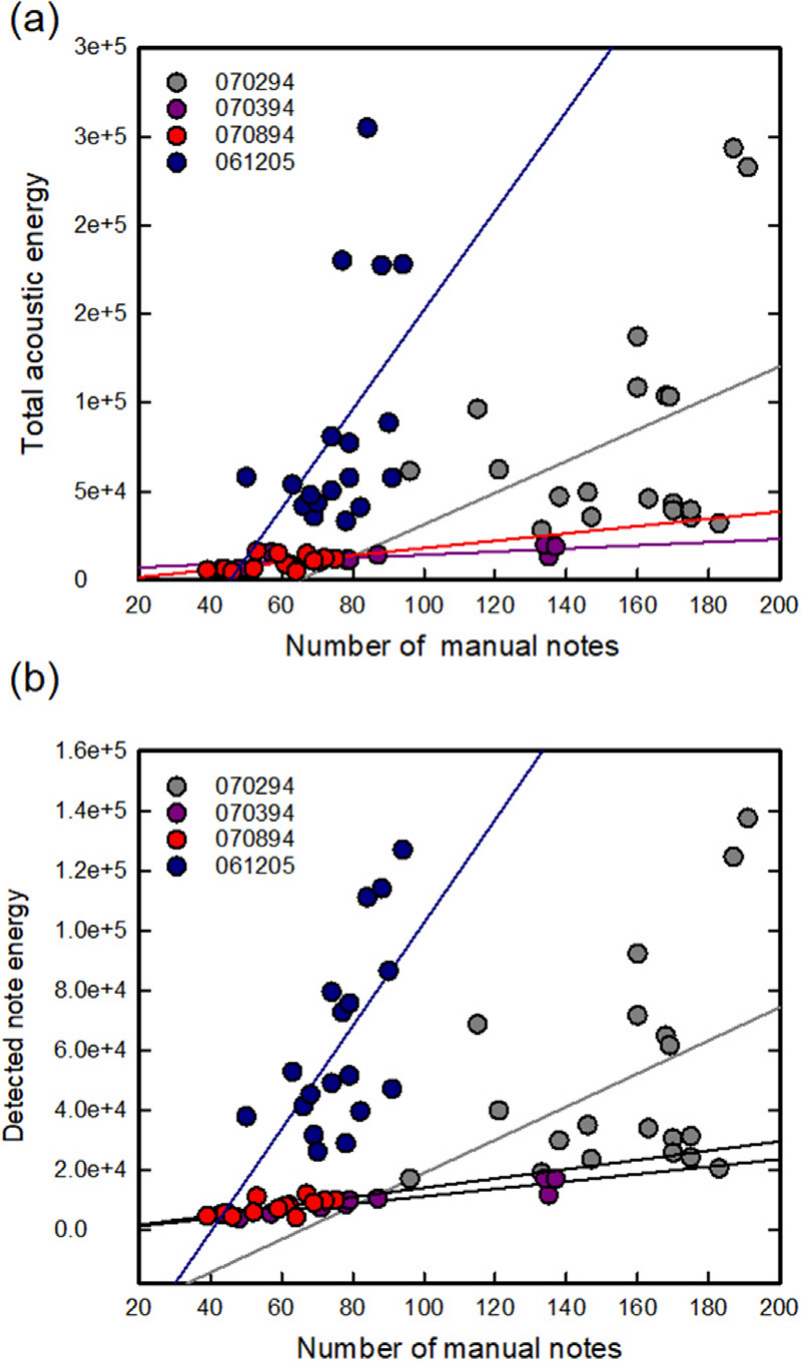
Results of the energy detection algorithm displayed as linear regressions. Data are from four recording nights (legends). (a) Relationship between number of manually counted notes and total acoustic energy (V^2^). Line indicates linear regression. (b) Relationship between number of manually counted notes and detected note acoustic energy (V^2^). Line indicates linear regression.

On two recording nights, there were significant relationships between numbers of manually counted notes and both total acoustic energy [070394: F(1,8) = 8.07, p = 0.025, r^2^ = 0.535; 061205: F(1,17) = 4.79, p = 0.044, r^2^ = 0.231, Fig. 2(a)] and detected note acoustic energy [070394: F(1,8) = 45.61, p < 0.001, r^2^ = 0.867; 061205: F(1,17) = 10.00, p = 0.006, r^2^ = 0.385, Fig. 2(b)]. On 070894, the relationship between manually counted notes and detected note acoustic energy was significant [F(1,15) = 13.87, p = 0.002, r^2^ = 0.498; Fig. 2(b)], but that with total acoustic energy was not [p = 0.14, r^2^ = 0.36; Fig. 2(a)]. For recording date 070294, the relationship between manually counted notes and either total acoustic energy (p = 0.246, r^2^ = 0.164) or detected note energy (p = 0.184, r^2^ = 0.209) did not reach statistical significance.

## 4. Discussion

Our results indicate that acoustic energy estimates can predict the number of advertisement call notes emitted by male bullfrogs vocalizing in small choruses, in spite of variation in calling activity across recording nights. To validate the energy method, we used as examples recordings from four sparse choruses with a small, known number of vocalizing bullfrogs located at variable distances from the recording microphone. For the total acoustic energy in each sample, which was calculated without detecting individual notes, we found significant relationships between the number of manually counted notes and acoustic energy for two of our four recording nights [Figs. 2(a) and 2(b)]. For the detected note acoustic energy, which relies on detection of energy in bounding boxes around individual notes, we found significant relationships with the number of manually counted notes on three of the four recording nights. Importantly, we found significant relationships between numbers of manually counted and automatically detected notes on all four recording nights, showing the usefulness of the automatic detection method.

These findings complement the work of Kloepper *et al.* (2016), who reported a significant relationship between the number of flying bats emerging from a cave and the corresponding acoustic energy in 1-s time windows. Here, we chose a longer time window for our analysis—5 min—because bullfrog notes are longer in duration and have longer and more variable call intervals than those of echolocating bats (Bee, 2004; Simmons, 2004). The length of the audio segment is an important variable that should be examined in further work.

Kloepper *et al.* (2016) investigated very dense groups of animals emerging from a fixed position. In addition, there was little anthropogenic noise at that recording site that could interfere with detection of the bat's ultrasonic calls. The results from our current study address these limitations but also suggest others. Bullfrog calling activity, as previously reported (Howard, 1978), is cyclical, varying over recording nights, and, as shown in our data, even within 5-min segments on the same recording night. This led to variation from night-to-night between the relationship of number of manually detected advertisement call notes and both measures of acoustic energy. In particular, we observed considerable variation in measures of total acoustic energy (Fig. 2), likely reflecting different levels of background noise, not eliminated by noise reduction, during recording nights. Because of this variation, reciprocal cross-validation, such as that conducted by Kloepper *et al.* (2016), was not possible using our dataset.

Even though we imposed a noise reduction algorithm on our audio samples, this was not sufficient to eliminate all sources of non-bullfrog sounds. Indeed, the frequency overlap between many anthropogenic noise sources and bullfrog advertisement calls makes such elimination challenging. Application of more sophisticated noise reduction algorithms (Sainburg and Gentner, 2021) may eliminate this problem. Although we observed good relationships between the number of manually detected and automatically detected notes (Table 1), the thresholding used in the energy detection algorithm (Fig. 1) eliminated notes from bullfrogs located far from the recording microphone. These notes were audible to human listeners and visible on spectrogram displays. As shown by Boatright-Horowitz *et al.* (2000), bullfrogs call preferentially to farther away compared to nearby males, highlighting the importance of spatial location for modeling chorus activity. The parameters of the bounding boxes used to define note energy were chosen to eliminate very short as well as long duration noise. Extraneous noise within the 0.2–2 s limits still could have been counted as bullfrog notes, producing greater numbers of automatically detected than manual detected notes (Table 1, 070394). In addition, the in-house program may not have picked up notes that occurred during intense portions of jet noise [Fig. 1(c)]. Aural and visual identification of notes can eliminate all anthropogenic noise, but this technique is time-consuming and unwieldly when studying dense choruses. Using multiple microphones around the chorus would provide a better measure of overall chorus activity and likely better fits with acoustic energy measures.

Our analyses focused on small choruses of 6–8 actively vocalizing male bullfrogs. Because of this small range in frog numbers, we could not compare reliably acoustic energy to the number of vocalizing animals, as was done by Kloepper *et al.* (2016) for dense swarms of echolocating bats. Rather, we aimed to assess whether our method could accurately predict the number of individual advertisement call notes. It is important to note that we quantified individual notes, not numbers of advertisement calls, which contain variable numbers of individual notes [from 2 to 12 (Capranica, 1965; Bee, 2004; Simmons, 2004)]. It is assumed that numbers of notes will co-vary with numbers of advertisement calls; quantifying this variation is feasible with manual analysis but more challenging with automated.

To apply the acoustic energy technique to census ponds, future work needs to record from ponds with higher densities of calling males. We also recommend a longer recording period to characterize the acoustic energy in a given pond. For sparse pond locations, using the detected note acoustic energy (after imposing a noise reduction algorithm) helps eliminate unwanted background noise and should result in better estimation of vocal activity and/or population density; at dense ponds, highly overlapping vocalizations should result in both detected note and total acoustic energy metrics equally estimating vocal activity and/or population.

Overall, these results indicate that bullfrog chorusing activity can be estimated using measures of acoustic energy. Moving forward, these approaches can be extended to census frogs with passive acoustics, even without needing to isolate individual notes. This method could be helpful not only in sparsely populated ponds, such as represented in this study, but also in ponds with dense populations of vocalizing frogs and overlapping vocalizations. This method also has the potential to census other vocal activities in patchy environments including nesting seabirds and soniferous reef fish.
